# Wetting characteristics of *Colocasia esculenta* (Taro) leaf and a bioinspired surface thereof

**DOI:** 10.1038/s41598-020-57410-2

**Published:** 2020-01-22

**Authors:** Manish Kumar, Rajneesh Bhardwaj

**Affiliations:** Department of Mechanical Engineering, Indian Institute of Technology Bombay, Mumbai, 400076 India

**Keywords:** Chemical engineering, Mechanical engineering, Fluid dynamics, Bioinspired materials

## Abstract

We investigate wetting and water repellency characteristics of *Colocasia esculenta* (taro) leaf and an engineered surface, bioinspired by the morphology of the surface of the leaf. Scanning electron microscopic images of the leaf surface reveal a two-tier honeycomb-like microstructures, as compared to previously-reported two-tier micropillars on a *Nelumbo nucifera* (lotus) leaf. We measured static, advancing, and receding angle on the taro leaf and these values are around 10% lesser than those for the lotus leaf. Using standard photolithography techniques, we manufactured bioinspired surfaces with hexagonal cavities of different sizes. The ratio of inner to the outer radius of the circumscribed circle to the hexagon (*b*/*a*) was varied. We found that the measured static contact angle on the bioinspired surface varies with *b*/*a* and this variation is consistent with a free-energy based model for a droplet in Cassie-Baxter state. The static contact angle on the bioinspired surface is closer to that for the leaf for *b*/*a* ≈ 1. However, the contact angle hysteresis is much larger on these surfaces as compared to that on the leaf and the droplet sticks to the surfaces. We explain this behavior using a first-order model based on force balance on the contact line. Finally, the droplet impact dynamics was recorded on the leaf and different bioinspired surfaces. The droplets bounce on the leaf beyond a critical Weber number (*We* ~  1.1), exhibiting remarkable water-repellency characteristics. However, the droplet sticks to the bioinspired surfaces in all cases of *We*. At larger *We*, we recorded droplet breakup on the surface with larger *b*/*a* and droplet assumes full or partial Wenzel state. The breakup is found to be a function of *We* and *b*/*a* and the measured angles in full Wenzel state are closer to the predictions of the free-energy based model. The *sticky* bioinspired surfaces are potentially useful in applications such as water-harvesting.

## Introduction

Superhydrophobic and hydrophobic surfaces have generated significant interest in the last two decades due to their potential technical applications in designing functional surfaces that exhibit properties such as self-cleaning, low drag, antifouling, water harvesting, anti-icing etc.^1–5^. In order to design such functional non-wetting surfaces, several researchers have been inspired by non-wetting characteristics of leaves of different species of plants. A remarkable example is a lotus leaf, that exhibits non-wetting characteristics due to the presence of micro- and nanoscale features on its surface. A notable review by Neinhuis and Barthlott^6^ compiled published data of wetting characteristics and morphology of leaves of around 200 plants. In particular, they attributed non-wetting characteristics to microstructures and wax present on the surface. Similarly, Koch *et al*.^7^ and Koch and Barthlott^8^ discussed diversity in the morphology of the surface of the leaves of the different plants and their role in determining the wetting characteristics. These reviews also discuss how to engineer or biomimic a surface for potential technical applications such as for self-cleaning, antifouling, reducing particle adhesion etc. In this context, Ma *et al*.^9^ studied the antifouling characteristics of a taro leaf using atomic force microscopy and attributed the antifouling to a two-tier structure on the leaf and in particular, to air trapped between second-tier nanostructures.

Previous reports show significant efforts made towards engineering non-wetting surfaces, inspired by the morphology of non-wetting leaves (for instance, see notable reviews by Guo *et al*.^2^, Liu *et al*.^3^ and Jiaqiang *et al*.^4^). Kim *et al*.^10^ developed a nanoimprinting technique to make a replica of a taro leaf. They reported static contact angles of 155 ± 3. 4° and 135 ± 5. 3° on the leaf and bioinspired surface, respectively. Liu *et al*.^11^ designed a mushroom-like doubly reentrant structures and demonstrated that such a surface is super-repellent to even low-energy liquids (e.g. a fluorinated solvent). Inspired by a lotus leaf, Frankiewicz and Attinger^12^ fabricated a metallic superhydrophobic surface using specific chemical processes. They demonstrated that a hydrophilic material like copper can be used to make a functional surface with a two-tier structure, which has similar non-wetting characteristics as the lotus leaf. Similarly, Patil *et al*.^13^ investigated wetting characteristics and droplet impact dynamics on surfaces with micropillars, manufactured by mimicking first-tier structure of the lotus leaf. They fabricated different surfaces by varying pitch of the pillars and investigated the role of impact velocity and the pitch. They concluded that by increasing the pitch, the non-wetting characteristic can be improved. However, at larger pitch and impact velocity, Cassie-Baxter to Wenzel wetting transition occurs. Malla *et al*.^14^ investigated the surfaces with rectangular microgrooves, bioinspired by a rice leaf. They varied the pitch of the grooves and droplet impact velocity, and reported different fates of the impacting droplet, namely, no-bouncing, partial bouncing and complete bouncing. Wang and Zhao^15^ and Wang *et al*.^16^ also experimentally investigated anisotropic wetting characteristics of corrugated and microgrooves surfaces, respectively. In these studies, they reported a strong dependence of the contact angle on the geometry of the grooves. Sharma *et al*.^17^ designed and replicated a surface inspired by leaf of *Gladiolus dalenii* plant. They mimicked the microstructure of the leave using soft lithography techniques and demonstrated an enhancement of 230% in water-harvesting on this surface. Ghosh *et al*.^18^ investigated wetting behavior and adhesion characteristics of a rose petal and resolved the liquid-substrate contact region using in-situ atomic force microscopy and confocal microscopy. They reported the Cassie impregnating wetting state on the rose petal, which does not let droplet to roll off from the surface. Very recently, Orejon *et al*.^19^ studied wetting and water vapor condensation characteristics on two-tier and three-tier engineered surfaces. The former surface shows sticky non-wetting behavior (similar to rose petal) and filmwise condensation while the latter showed non-wetting (similar to lotus leaf) and dropwise condensation. They attributed non-wetting characteristics of the three-tier surface to its nanoscale features (third-tier). In addition, previous studies also reported the fabrication of non-wetting surfaces bioinspired by the exoskeleton of insects. For instance, bioinspired by springtail cuticle, Zhu *et al*.^20^ designed and fabricated an omniphobic surface using a microfluidic emulsion templating technique.

The brief literature survey shows that several studies reported wetting characteristic of lotus leaf and bioinspired surfaces based on it. Several studies also targeted useful applications mentioned earlier and quantified the effectiveness of such functional surfaces for these applications. The surfaces with structures similar to the morphology of the lotus leaf i.e., two-tier or three-tier micropillars exhibit excellent non-wetting (superhydrophobic) characteristics. Here, we report wetting characteristics on a taro leaf that does not exhibit conventional micropillars (e.g. on a lotus leaf) and the morphology of the taro leaf shows a two-tier structure of hexagonal microcavities i.e., honeycomb-like structure. While the static contact angle on the taro leaf was reported by Kim *et al*.^10^, a detailed morphology of the surface of the leaf, contact angle hysteresis and droplet impact dynamics on the leaf have not been reported in the literature, to the best of our knowledge.

In the context of the bioinspired surfaces based on the taro leaf, Ma *et al*.^9^ used an nanoimprinting technique to make a replica of the leaf to study antifouling characteristics of the surface. However, to the best of our knowledge, there has not been any attempt reported to fabricate an engineered surface based on the taro leaf using *bottom-up* approach. This approach is particularly suited to understand the role of micro- and nanoscale structures in determining wettability and associated droplet impact dynamics on a bioinspired surface. Therefore, the second objective of this paper is to fabricate a bioinspired surface based on taro leaf and understand these characteristics. With the help of measurements and a theoretical model, here we show how the dimensions of a honeycomb-like structure influence the wettability and associated droplet impact dynamics of the bioinspired surface. The present study also shows a key difference between the bioinspired surfaces based on the two morphologies, namely, micropillars-like and honeycomb-like structure. The former exhibit a discontinuous contact line as compared to that for a droplet on a smooth surface^21^ and consequently, it results in a low contact angle hysteresis. The latter exhibits a continuous contact line and consequently, the surface shows a very large contact angle hysteresis. We explain it using the help of a first-order model why a honeycomb-like surface exhibits a large hysteresis despite being superhydrophobic.

## Results

### Wetting characteristics

#### Taro leaf

**Figure 1:**
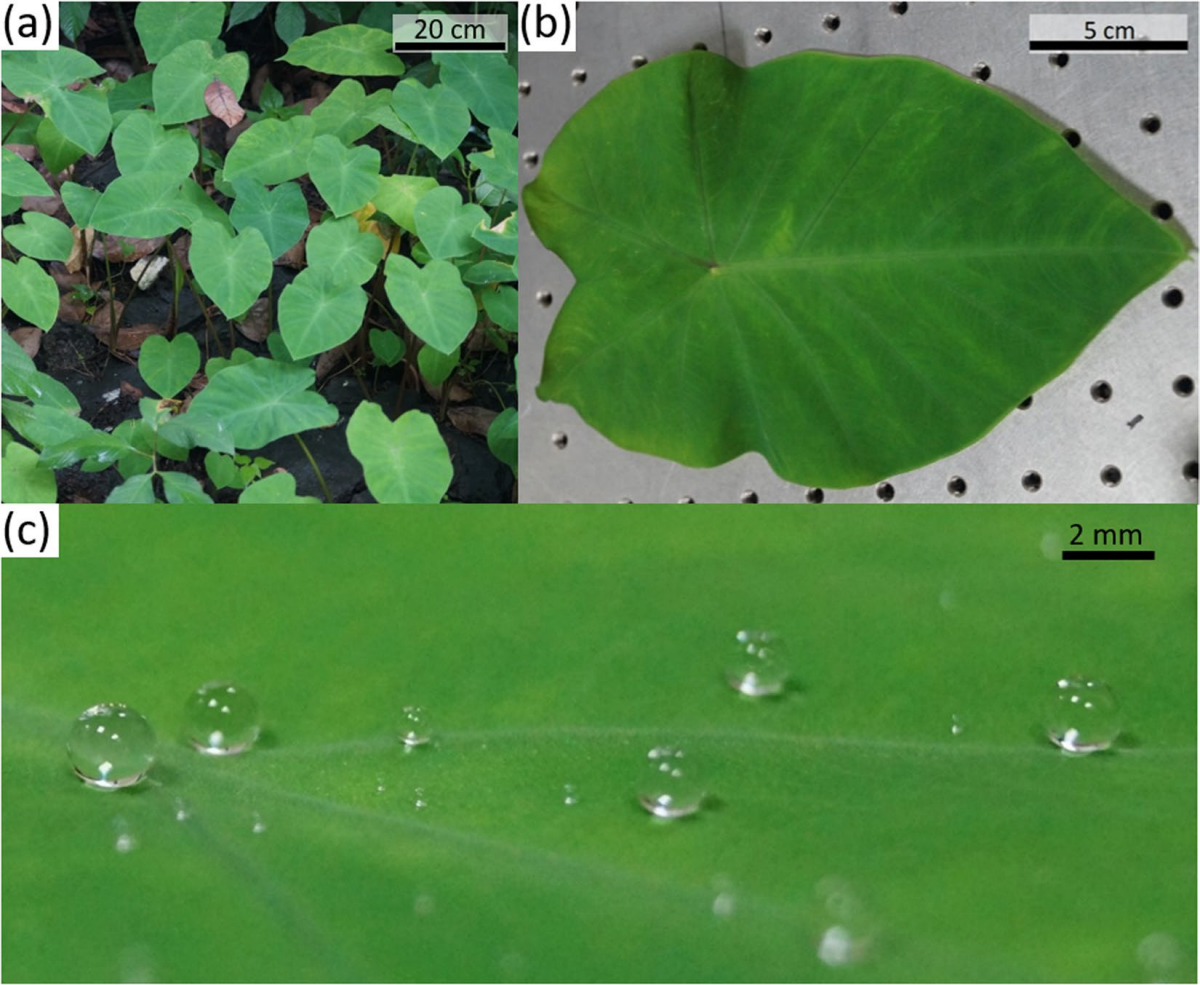
(**a**) A photograph of *Colocasia esculenta* (taro) plants in garden of our campus. Some leaves of an other plant (bottom left corner) can be seen as wet as compared to the leaves of the taro. (**b**) A plucked leaf of taro (**c**) Deionized water droplets on the leaf.

Figure 1a,b show photographs of leaves of the taro plant before and after plucking, respectively. Additional images of the leaves before plucking are provided in supplementary information. Figure 1c shows sessile water droplets on the leaf, qualitatively exhibiting the the superhydrophobicity of the leaf. To quantify the wetting characteristics of the leaf, we measured the static contact angle, advancing angle, and receding angle of a water droplet on the leaf. We repeated the experiments around seven times at different locations of the same leaf and on different leaves for different volumes of droplets in the range of [2.1–3.9] *μ*L. These measurements are listed in Table 1. The average value of the static contact angle, advancing angle and receding angle are around 150 °, 153 ° and 144 °, respectively, implying contact angle hysteresis of around 9 °. The maximum uncertainty in these measurements is around ± 2 °. Therefore, the leaf shows excellent superhydrophobic characteristics. The rear side of the leaf also shows similar wetting characteristics and these measurements are provided in the supplementary﻿ information.

**Table 1:** Measured values of static,advancing and receding contact angle on the taro leaf.

Volume (*μ*L)	Static contact angle (°)	Advancing contact angle (°)	Receding contact angle (°)
2.1	150.9	155	142
2.4	149.5	153	144
2.6	152.2	152	144
2.8	152.8	155	147
3.2	149.0	151	142
3.2	150.2	154	145
3.9	149.4	154	145
Average values	150.5	153.4	144.1
Standard deviation	1.5	1.5	1.8

**Figure 2:**
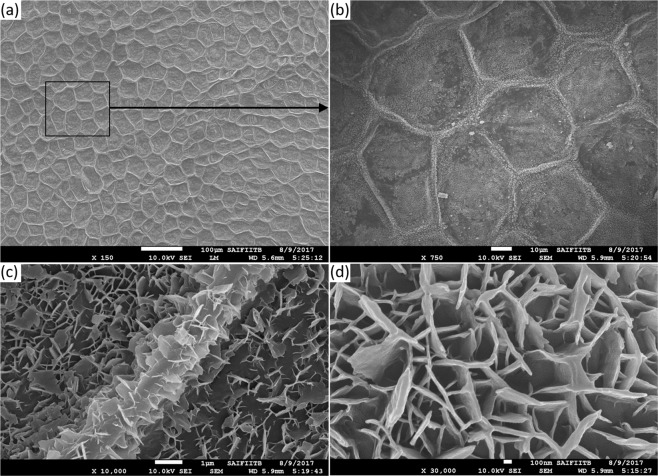
SEM image of the surface of the taro leaf. Zoomed-in view of the SEM image with systematic increase in magnification (**a**) 150X (**b**) 750X (**c**) 10000X (**d**) 30000X.

Figure 2 shows SEM images of the surface of the leaf at four different increasing zoom levels from (a) to (d). Additional SEM and optical microscopic images are provided in the supplementary information. In Fig. 2a,b we note pentagon as well as hexagon structures creating honeycomb-like structures on the surface of the leaf. In Fig. 2c,d we see a second tier flakes-like nanoscale structures on top of the primary pentagon and hexagon structure. Therefore, the leaf exhibits a two-tier structure, honeycomb structure as the primary structure and flakes-like structure as the secondary structure. The average side of the polygon is around 50 *μ*m, and the thickness of the wall of the hexagon is around 3 *μ*m.

#### Bioinspired surface

**Figure 3:**
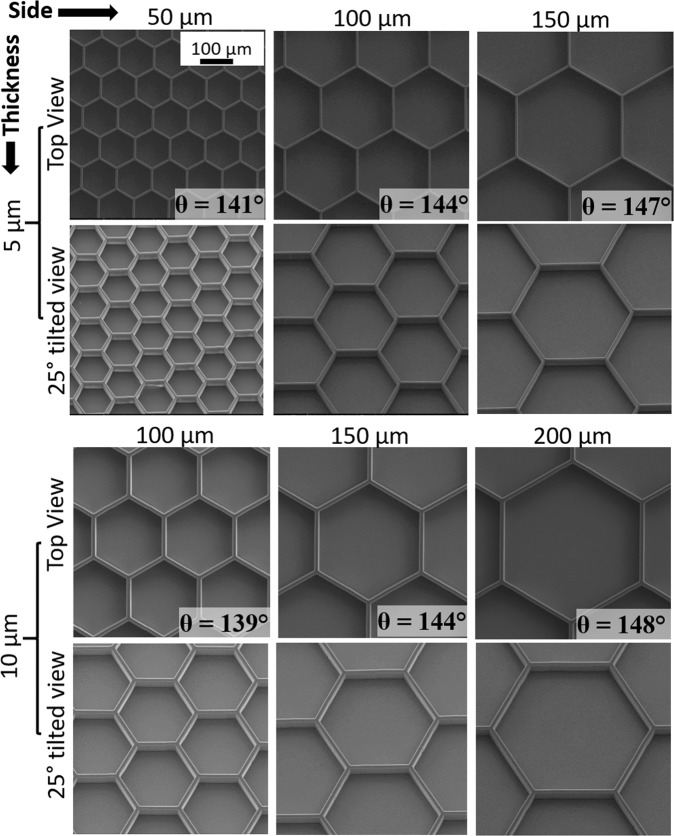
SEM images of the bioinspired surface taken from top and 25 ° tilt. The first and third row show the surfaces manufactured with different lengths of the side of the hexagonal cavity with 5 *μ* and 10 *μ* thickness of the wall of the cavity, respectively. The length of the side is shown on the top of each frame in first and third rows.

*Measurement of static contact angle*: Figure 3 shows SEM images of bioinspired surfaces manufactured in the cleanroom, with different thicknesses and sides of the hexagon. The top and 25 ° tilted view, are shown. Additional microscopic images are provided in the supplementary﻿ information. The measured static contact angle for a water droplet is indicated on each top-view frame. Figure 3 shows that on increasing the side of the hexagon while keeping the thickness constant, the static contact angle increases. However, if the thickness is increased, keeping the side constant, the contact angle decreases. By varying the side or thickness of the hexagon, fractional area of contact between the droplet and surface changes for a droplet in Cassie-Baxter state. As the fractional area decreases, the contact angle increases and vice-versa. The maximum static contact angle (*θ*) achieved on the bioinspired surface is around 148 ° for the hexagon thickness of 10 *μ*m and side 200 *μ*m.

**Figure 4:**
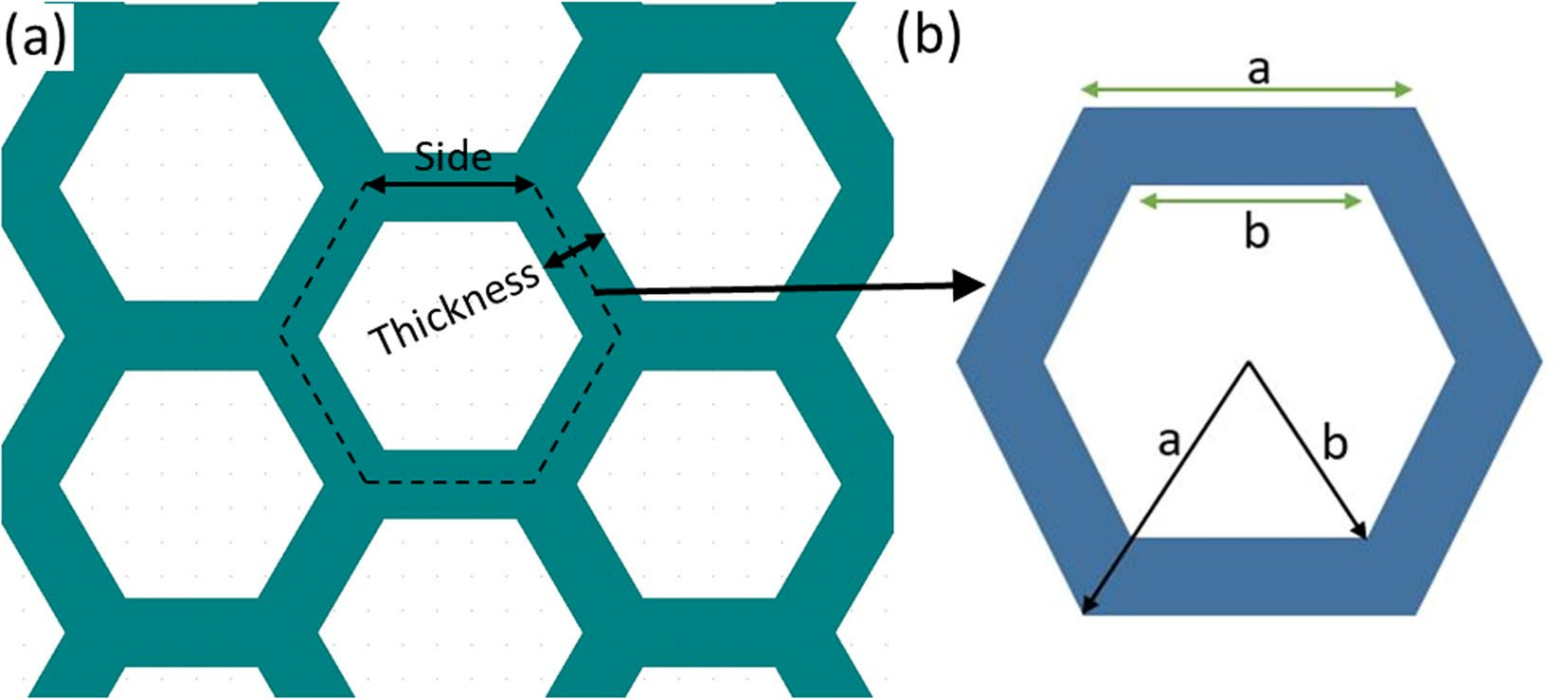
(**a**) Schematic of honeycomb geometry of the bioinspired surface, biomimicking first-tier structure found on the taro leaf (**b**) A unit cell of the honeycomb structure showing geometrical parameters *a* and *b*. AUTOCAD–R24.0 (www.autodesk.com) was used to create the CAD model.

We collapse the data by defining a geometrical parameter *b/a* that is the ratio of inner to the outer radius of the circumscribed circle to the hexagonal cavity (Fig. 4a,b). In other words, *b/a* represents the relative thickness of hexagonal wall with respect to length of the side of a hexagonal unit. A larger value of *b/a* denotes a smaller thickness of the hexagonal wall and vice-versa. *b*∕*a* = 1 for a hypothetical case of zero thickness wall. Table 2 summarizes the surfaces tested with different geometries and the corresponding values of *b*∕*a* and *θ* and shows that *θ* monotonically increases with *b*∕*a*.

**Table 2:** Comparison between measurements and predictions of a free-energy based model, proposed by Patankar^22^, assuming that the droplet is in Cassie-Baxter state.

S. No.	Side, a	Thickness	b	*b/a*	Contact angle	Cassie theory
	(*μ*m)	(*μ*m)	(*μ*m)		*θ* (°)	${\theta }^{c}$ (°)
1	50	30	32.7	0.65	104.1	111.3
2	50	20	38.5	0.77	116.7	123.1
3	80	30	62.7	0.78	115.8	124.8
4	80	20	68.5	0.86	121.1	134.6
5	120	30	102.7	0.86	120.4	134.6
6	120	20	108.5	0.90	126.8	142.8
7	50	5	47.1	0.94	141.9	151.1
8	100	10	94.2	0.94	139.8	151.1
9	150	10	144.2	0.96	144.2	156.3
10	100	5	97.1	0.97	144.8	151.1
11	200	10	194.2	0.97	148.6	159.5
12	150	5	147.1	0.98	147.7	163.3

**Figure 5:**
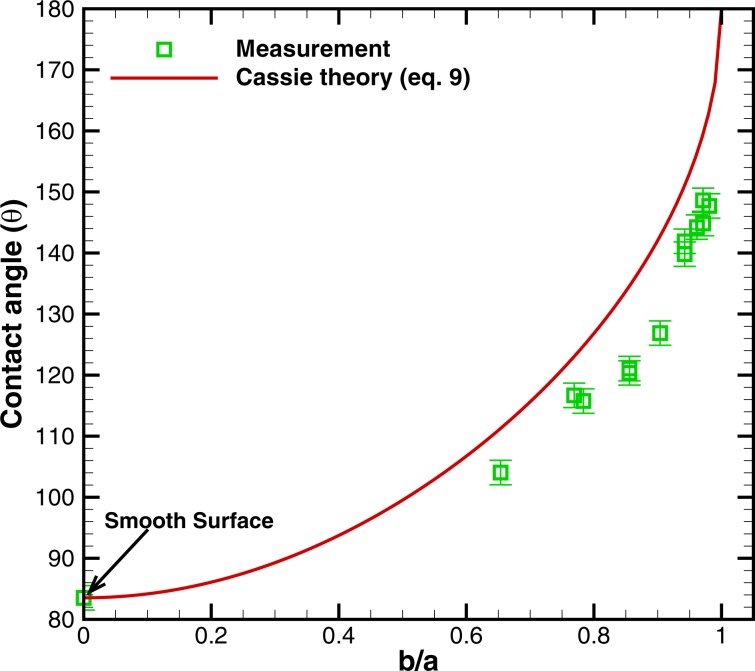
Comparison between measured static contact angles at different values of *b*/*a* on the bioinspired surfaces and the predictions of a free-energy based model for a droplet in Cassie-Baxter state. The model was proposed by Patankar^22^ and here we employ it for the surfaces with hexagonal-cavities. *b*/*a* is the ratio of inner to the outer radius of the circumscribed circle to the hexagonal cavity.

*Comparison of measurements with a theoretical model*: Figure 5 shows the comparison of the measured static contact angles (*θ*) of a water droplet with the predictions of the model for Cassie-Baxter state, described in ‘Methods’. The measurements as well as the model predictions are plotted against *b*∕*a*. Figure 5 shows that *θ* increases with *b/a* in measurement as well as in the model. The data is in reasonably good agreement, with the model capturing the trend of the measurements. The maximum error in the values predicted by the model with respect to measurement is around 10%. This also implies that a gently placed droplet on the bioinspired surfaces assumes Cassie-Baxter state.

**Figure 6:**
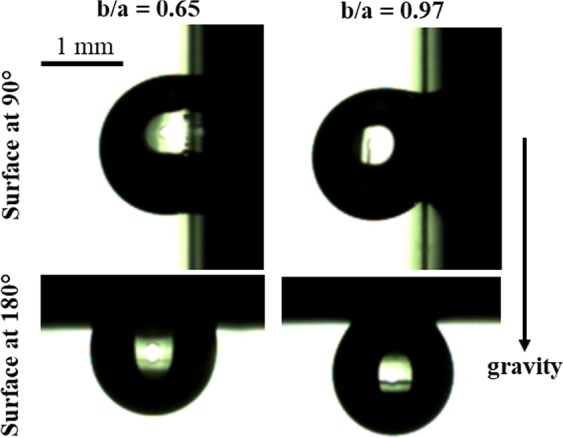
Test of droplet sliding on the bioinspired surfaces with *b*/*a* = 0.65 (left) and *b*/*a* = 0.97 (right). Images for the tilt at 90° and 180° are shown in top and bottom row, respectively. The droplet does not slide and sticks to the surface even for the tilt at 180°.

*Contact angle hysteresis*: We used sliding droplet method to measure advancing and receding contact angle on the bioinspired surfaces. In these measurements, the droplet did not slide on any bioinspired surface at any tilt angle, and it got stuck to the surface. Figure 6 shows this behavior on two surfaces, with a smaller and larger *b/a* considered (*b/a* = 0.65 and 0.97). In case of tilt at 90 °, the droplet loses its symmetry due to gravity and the contact angle at the leading edge of the contact line is larger than at the trailing edge (Fig. 6, first row). In case of tilt at 180°, the droplet retains its spherical cap shape and does not fall off the surfaces i.e., it becomes a pendant droplet (Fig. 6, second row).

**Figure 7:**
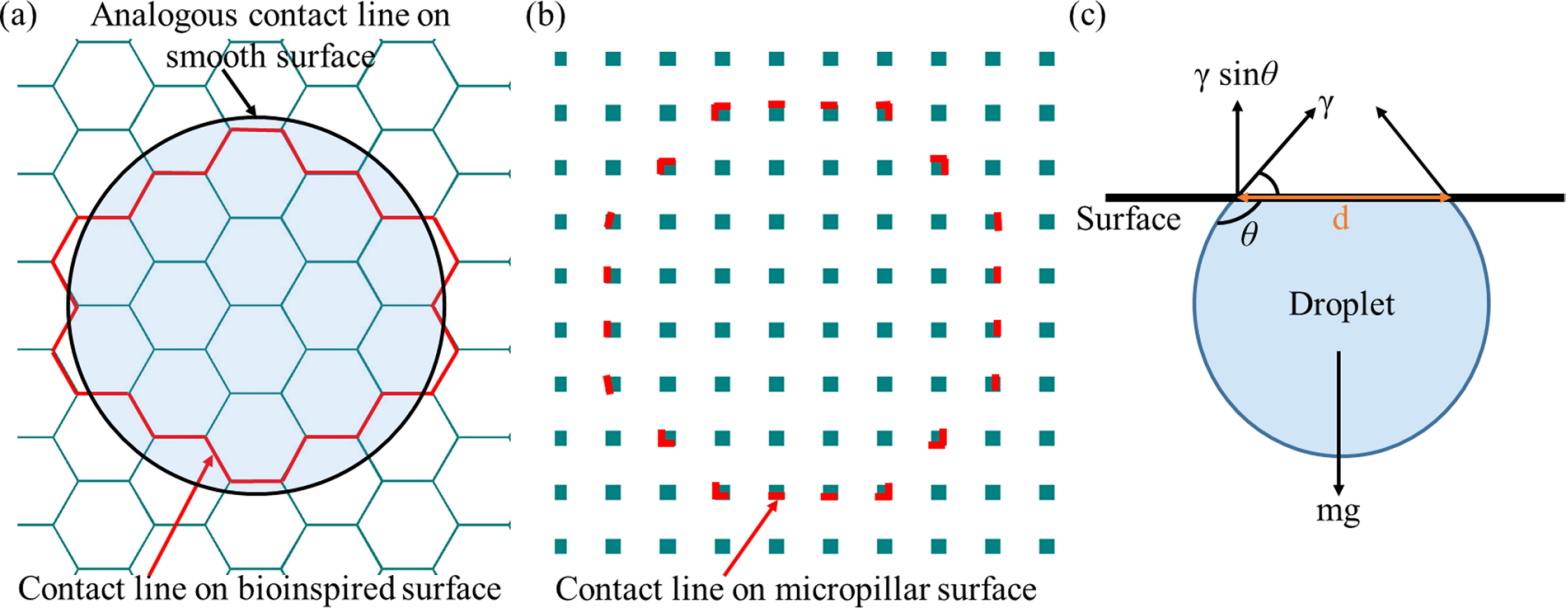
(**a**) The contact line (shown as red) on the bioinspired surface with hexagonal cavities. The contact line can be approximated as a circle, as shown in the figure (black circle). (**b**) The contact line on a surface with micropillars. (**c**) Free body diagram of a pendant droplet with possible forces shown on the droplet. The droplet sticks to the bioinspired surface if the vertical component of the surface tension force acting on the contact line exceeds the droplet weight.

In order to explain this behavior, we hypothesize that the continuous contact line is responsible for the *sticking* of the droplet on the surface, By contrast, the contact line is discontinuous on a micropillared surface, as shown in Fig. 7b. We estimate the order of magnitude of surface tension force (*F*_*γ*_) and gravitational force (*F*_*g*_ i.e., droplet weight) of a pendant droplet (tilt at 180 °) on honeycomb and micropillared surfaces, as shown schematically in Fig. 7c. Since the pendant droplet represents the case in which it is most likely to fall off the surface, as compared to the surfaces titled at lesser than 180 °, we present the analysis for the pendant droplet. The order of *F*_*g*_ of a typical 2.57 *μ*L droplet is given by:

1 $${F}_{g}=\rho Vg=2.52\times 1{0}^{-5}N \sim 1{0}^{-5}$$

where *ρ* is the density of water (998 kgm^−3^), *V* is the droplet volume, and *g* is the gravitational acceleration (9.81 ms^−2^).

In order to estimate *F*_*γ*_, we approximate zig-zag contact line on the bioinspired surface, shown in red as circle in Fig. 7a. The perimeter of the circle is slightly shorter than the actual length of the contact line. The expression and order of magnitude *F*_*γ*_ for a bioinspired surface (*b/a* = 0.98) is given as follows:

2 $${F}_{\gamma }=\pi d\gamma sin\theta =1.44\times 1{0}^{-4}N \sim 1{0}^{-4}$$

where *γ* is surface tension (7.2 × 10^−2^ Nm^−1^), *d* is the wetted diameter and *θ* is the contact angle (*θ* = 139 ° for a pendant droplet on the surface with *b*∕*a* = 0.98). Assuming droplet shape as a spherical cap, we estimate *d* with the given droplet volume (2.57 *μ*L) and contact angle and obtain *d* ≈ 1 × 10^−3^ mm. Therefore, in the case of the bioinspired surface (continuous contact line surface), the order of magnitude of supporting surface tension force (*F*_*γ*_) is one order of magnitude larger than the weight of the droplet (*F*_*g*_). Therefore, the surface tension force arrests the contact line motion on these surfaces, explaining the sticking behavior of the droplet and consequently, it results in the largest possible contact angle hysteresis.

In case of a discontinuous contact line, i.e., on a micropillared surface (Fig. 7b), we consider a surface with pitch (*p*) of 76 *μ*m and cross-section area of the pillar as square with *a* = 20 *μ*m side, considered by Patil *et al*.^13^. The measured static contact angle on this surface is around 150°^13^. We consider the same droplet volume (2.57 *μ*L) on this surface, as done earlier on the honeycomb surface. Approximate number of pillars (*n*) on the contact line is given by *πd*/*p* i.e., around 41. Thus, the approximate length of the contact line is, *l*_*c*_ ≈ *na* = 0.82 mm. The expression and order of magnitude *F*_*γ*_ on the micropillared surface is given as follows:

3 $${F}_{\gamma }={l}_{c}\gamma sin\theta =3.04\times 1{0}^{-5}N \sim 1{0}^{-5}$$

Therefore, from Eqs. 1 and 3, *F*_*γ*_ ~ *F*_*g*_ on the micropillared surface,. This implies that the droplet slides on the micropillared surface, consistent with the measurements of Patil *et al*.^13^.

**Figure 8:**
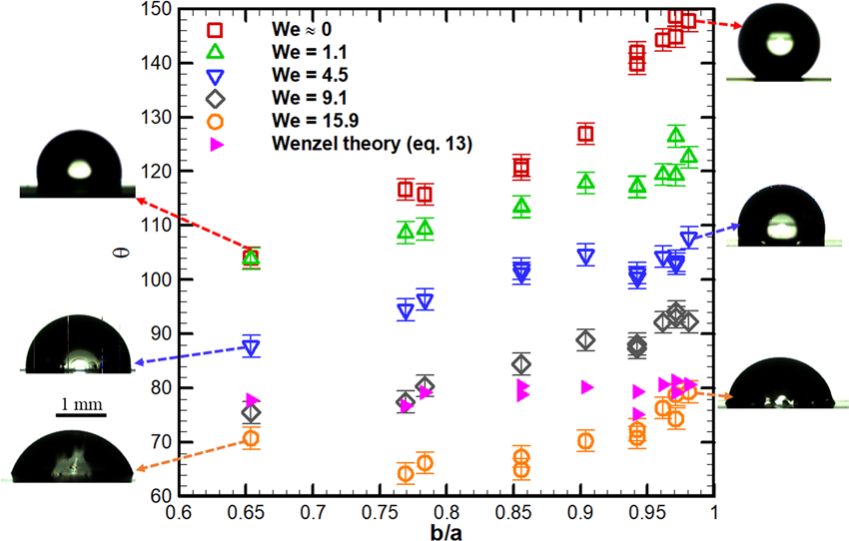
Static contact angle of the droplet (*θ*) on the bioinspired surface measured after it impacts on the surface with a given Weber number (*We*) and becomes sessile. The angle is plotted as a function of *b*/*a* i.e., the ratio of inner to the outer radius of the circumscribed circle to the hexagonal cavity. Different cases of *We* are considered and *We* ≈ 0 corresponds to a gently deposited droplet. Predictions of *θ* obtained by a free-energy based model are also plotted in which droplet is assumed to be in full Wenzel state. Left and right insets represent sessile droplet shapes for *We* ≈ 0, *We* = 4.5 and *We* = 15.9, marked by color coded arrows.

*Cassie-Baxter to Wenzel state wetting transition*: We estimate impact velocity or Weber number (*We*) for Cassie-Baxter to partial or full Wenzel state wetting transition on the bioinspired surfaces. We carried out measurements at several impact velocities or *We*, as given in ‘Methods’. The droplet becomes sessile shortly after the impact, and we measured the static contact angle of the sessile droplet (*θ*) using Eq. 4. Figure 8 shows the variation of *θ* with *b/a*, for different cases of *We*. *We* ≈ 0 corresponds to a gently placed droplet on the surface.

**Figure 9:**
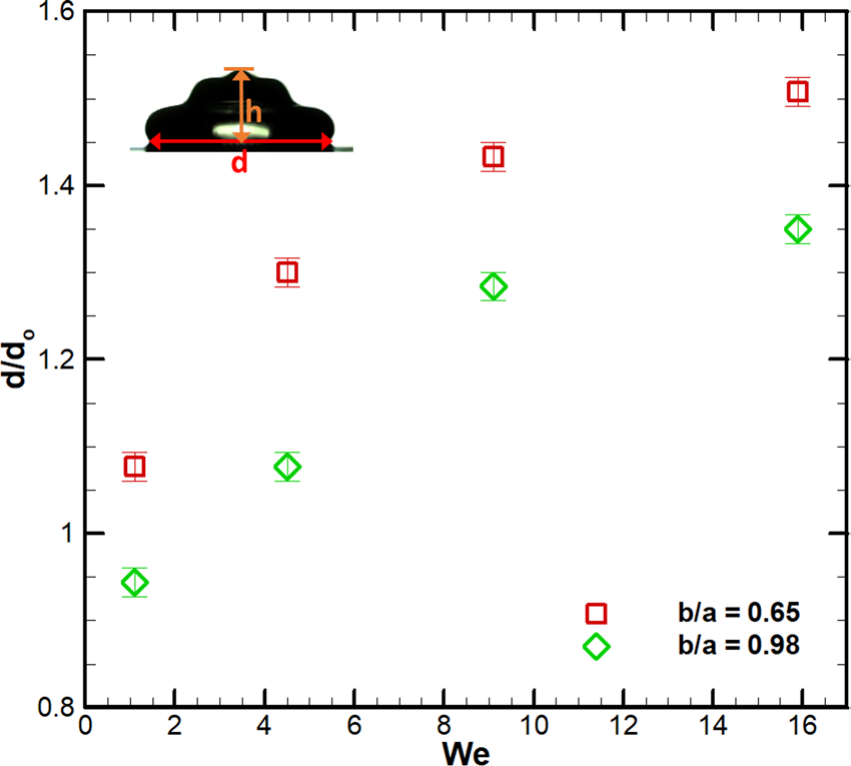
Variation of dimensionless wetted diameter of the droplet (*d*/*d*_0_) in the sessile state with respect to Weber number (*We*) for two cases of *b/a* = 0.65 and 0.98.

In Fig. 8, *θ* increases with *b/a* for all cases of *We*. The largest increase is for *We* ≈ 0, explained by an increase in the hydrophobicity of the surface with *b/a* while the droplet assumes Cassie-Baxter state, as discussed earlier. However, *θ* decreases with an increase in *We* at a given *b/a*. We attribute this behavior by postulating that the droplet assumes partial or full Wenzel state during the impact. Note that if the droplet were in Cassie-Baxter state, *θ* would not have changed with *We*. To verify this hypothesis, we plot the dimensionless wetted diameter (*d*/*d*_*o*_) in the sessile state as function of *We* in Fig. 9. Figure 9 illustrates that at a given *We*, *d*/*d*_*o*_ is larger for *b*/*a* = 0.65 as compared to *b*/*a* = 0.98, explained by a larger hydrophobicity of the former case. We note that as *We* increases at a given *b*/*a*, *d*/*d*_*o*_ increases. If the droplet were in Cassie-Baxter state, *d*/*d*_*o*_ would not have changed with *We*. Therefore, the droplet spreads more due to a larger kinetic energy available at larger *We*. However, the contact line motion gets arrested since the droplet assumes a partial or full Wenzel state. This implies a larger *d*/*d*_*o*_ in the sessile state at larger *We* (Fig. 9) and consequently, it reduces the corresponding *θ* (Fig. 8). A larger *We* (or impact velocity) corresponds to a larger break-in pressure^12,14^, which helps to break the liquid-gas interface in the microcavities and consequently, the droplet fills those cavities to assume a partial or full Wenzel state. The height of the wall of the cavity is large enough (29 ± 1 *μ*m) to avoid the wetting transition by sagging of the liquid-gas interface above the cavity^14^.

To further corroborate our measurements, we plot *θ* in Wenzel state predicted by a free-energy based model^22^, described in ‘Methods’. The values estimated using Eq. 13 are plotted together with measurements at several *We* as function of *b*/*a* in Fig. 8. At *We* ≈ 0, the droplet is in Cassie state and therefore, the predicted values of Wenzel state do not agree with measurements at *We* ≈ 0. The model predictions are much closer to *We* = 9.1 and *We* = 15.9, with a maximum error of around ±16%. The errors are larger for *We* = 1.1 and *We* = 4.5. This is explained by the fact that droplet could assume partial Wenzel state at intermediate *We*. However, the model considers a full Wenzel state. Overall, the Cassie-Baxter to Wenzel state wetting transition depends on the *We* (or impact velocity) and *b*/*a* (shape of the cavity).

### Droplet impact dynamics

#### Taro leaf

**Figure 10:**
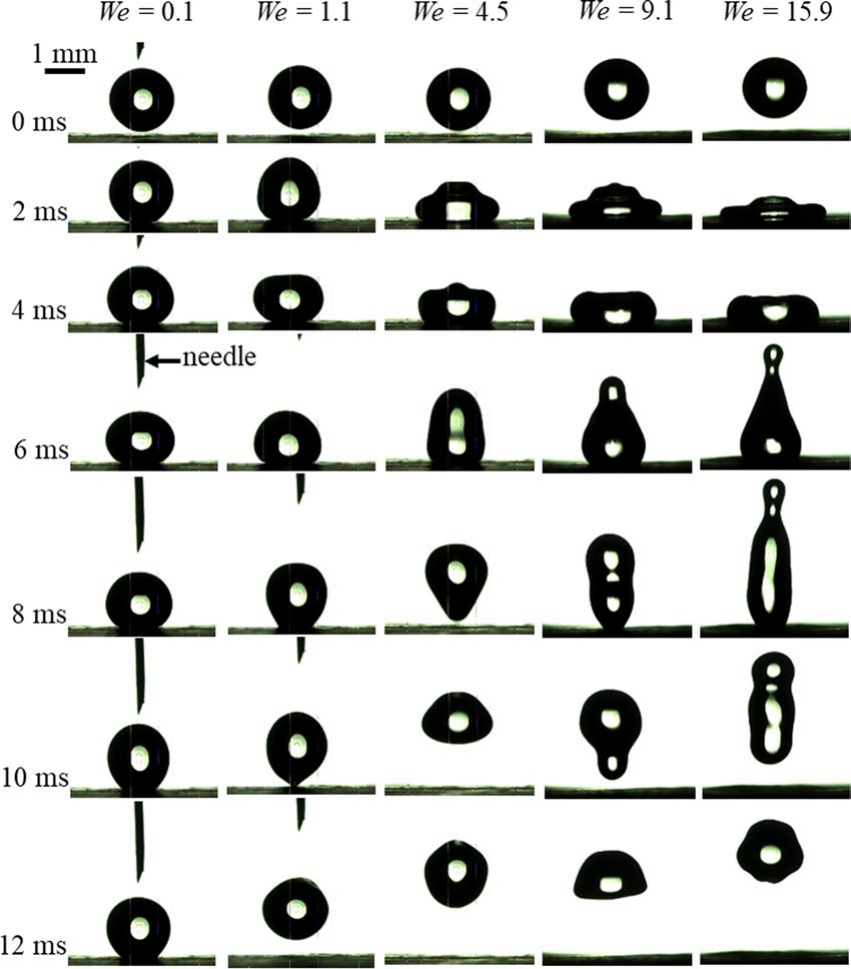
Impact dynamics of a microliter water droplet on the taro leaf at different Weber numbers (*We*) or impact velocities. The time instances of each row is indicated on the left. High-speed visualization movies are provided with the supplementary information. Supplementary information 9, 10, 11 and 12 correspond to cases of *We* = 1.1, 4.5, 9.1 and 15.9, respectively.

To understand water repellency characteristics of the leaf, we recorded the impact dynamics of a water droplet on it at several impact velocities. These velocities and corresponding Weber numbers (*We*) are provided in ‘Methods’. Figure 10 shows the impact dynamics on the leaf at different *We*. High-speed visualization movies of these cases are provided with the supplementary﻿ information. Each column in Fig. 10 shows time-sequenced frames of the impacting droplet for different cases of *We*, just before the impact (0 ms) and after the impact till 12 ms. For all cases of *We* except the lowest (*We* = 0.1), the impact dynamics is nearly similar. First, the droplet spreads and wets the surface with the maximum spreading. Second, the droplet rebounds with a receding contact line and simultaneously stretches in the vertical direction. Finally, the droplet detaches from the surface.

As *We* increases, the droplet spreading is faster (scales as *d*_*o*_/*v*) and the instantaneous maximum wetted diameter of the droplet increases. This is evident in the second row of Fig. 10 at 2 ms. The vertical stretching before the detachment of the droplet from the surface increases with an increase in *We*. During the impact, the kinetic energy of the droplet converts to surface energy and vice-versa during the recoiling. The droplet bounces if during the rebound the sum of kinetic and surface energy exceeds the initial surface energy^23,24^. Since the taro leaf exhibits superhydrophobicity, a larger kinetic energy is available during the recoiling, which is responsible for the bouncing.

Out of the five cases of *We*, the droplet bounces for each *We* except at *We* = 0.1. Theoretical minimum velocity at which bouncing starts to occur is expressed as follows^24,25^, ${v}_{c} \sim \sqrt{\gamma | cos{\theta }_{a}-cos{\theta }_{r}| /(\rho {d}_{o})}$. Substituting measured values of *θ*_*a*_ and *θ*_*r*_ (Table 1) in this expression, we obtain *v*_*c*_ = 0.062 m/s. In present measurements, the droplets start to bounce at 0.22 m/s, consistent with the theoretical model. Overall, the leaf exhibits excellent water repellency characteristics.

#### Bioinspired surface

**Figure 11:**
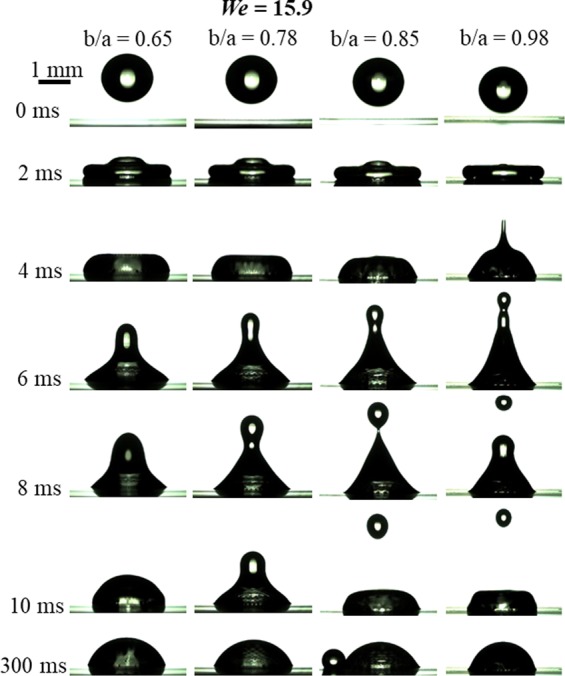
Impact dynamics of a microliter water droplet on bioinspired surfaces with different values of *b/a*, keeping Weber number as constant (*We* = 15.9, i.e., *v* = 0.82 m/s). High-speed visualization movies are provided with the supplementary information. Supplementary information 1, 2, 3 and 8 correspond to cases of *b*/*a* = 0.65, 0.78, 0.85 and 0.98, respectively.

Figure 11 shows the impact dynamics of the droplet at different values of *b/a* keeping *We* constant at 15.9. High-speed visualization movies of these cases are provided with the supplementary information. The droplet does not bounce in all cases of *We*, however, it breaks up at larger *b/a*. As *b/a* increases, the droplet fate changes from *no-breakup* (*b/a* = 0.65, 0.78) to *breakup* (*b/a* = 0.85, 0.98). In the latter, the droplet rebounds, *necking* of the droplet occurs (as seen in row corresponding to 6 ms in Fig. 11) and it does not bounce. A secondary smaller size droplet (4-8% volume of the primary droplet) detaches from the primary droplet in cases of *breakup*. Comparing frames in row corresponding to 8 ms in Fig. 11, we note that a transition from no-breakup to breakup occurs as *b/a* increases. The last row of the Fig. 11 shows that the droplet becomes sessile at around 300 ms after the impact.

These trends can be explained as follows. As the droplet spreads, the available kinetic energy converts into the surface energy and at the instance of maximum spreading, most of the kinetic energy converts into the surface energy. After the maximum spreading, the droplet contact line pins at the edge of the wall of the hexagonal cavity. Similar behavior of the pinning of the contact line was reported on a corrugated surface and surface with protruded sharp edges, by Wang and co-workers^15,26^. While the droplet rebounds, the surface energy converts into the kinetic energy. As *b/a* increases, the hydrophobicity of the surface increases (Table 2), that helps in reducing the maximum spreading. This is due to the fact that the increase in the surface energy at the expense of the kinetic energy at the instance of the maximum spreading is smaller on a surface with larger hydrophobicity^24^. In Fig. 11, the dimensionless wetted diameter at the instance of maximum spreading (*d*∕*d*_*o*_) for *b/a* = 0.98 is around 10% lesser than at *b/a* = 0.65 (Fig. 9). Therefore, lesser kinetic energy lost into surface energy and larger kinetic energy is available during the recoil at larger *b*∕*a*. During recoiling, the available kinetic energy squeezes the droplet upwards due to the pinned contact line and consequently, a liquid column rises upward around axis of impact. Since the kinetic energy dominates surface energy, the droplet undergoes breakup at larger *b*/*a*.

**Figure 12:**
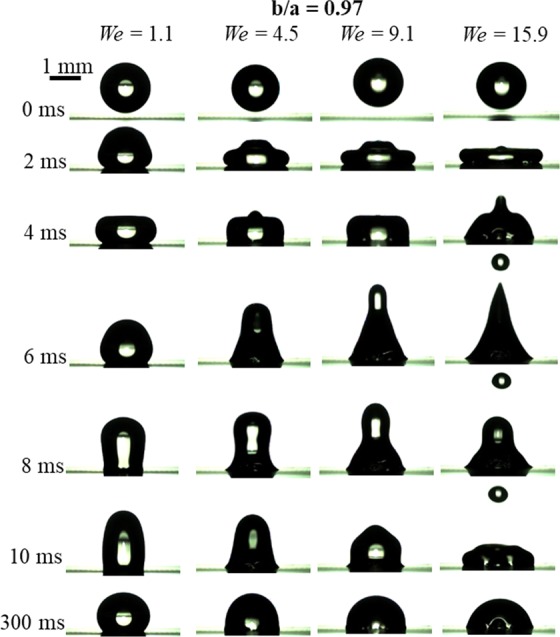
Impact dynamics of a microliter water droplet on a bioinspired surface with *b/a* = 0.97, at different Weber numbers (*We*) or impact velocities. High-speed visualization movies are provided with the supplementary information. Supplementary information 4, 5, 6 and 7 correspond to cases of *We* = 1.1, 4.5, 9.1 and 15.9, respectively.

**Figure 13:**
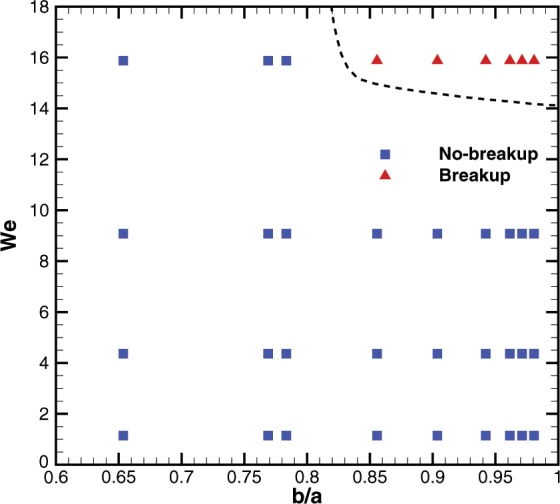
Regime map of the droplet fate on the bioinspired surfaces, namely, no-breakup and breakup, on Weber number (*We*) - *b*/*a* plane. A dashed line is shown as guide to the eye. The break-up is defined if the volume of the secondary droplet is more than 4% of the initial volume.

Similarly, Fig. 12 shows the effect of *We* on the impact dynamics keeping *b/a* constant (= 0.97). High-speed visualization movies of these cases are provided with the supplementary information. As *We* increases, the droplet fate changes from no-breakup (*We* = 1.1 to 9.1) to breakup (*We* = 15.9). As *We* increases, the available kinetic energy during the recoiling increases and it helps in neck formation and the subsequent breakup. A regime map is plotted on *b*/*a*-*We* plane in Fig. 13 and summarizes the droplet fate. The map shows that the breakup occurs at *We* = 15.9 and *b/a* > 0.85.

**Figure 14:**
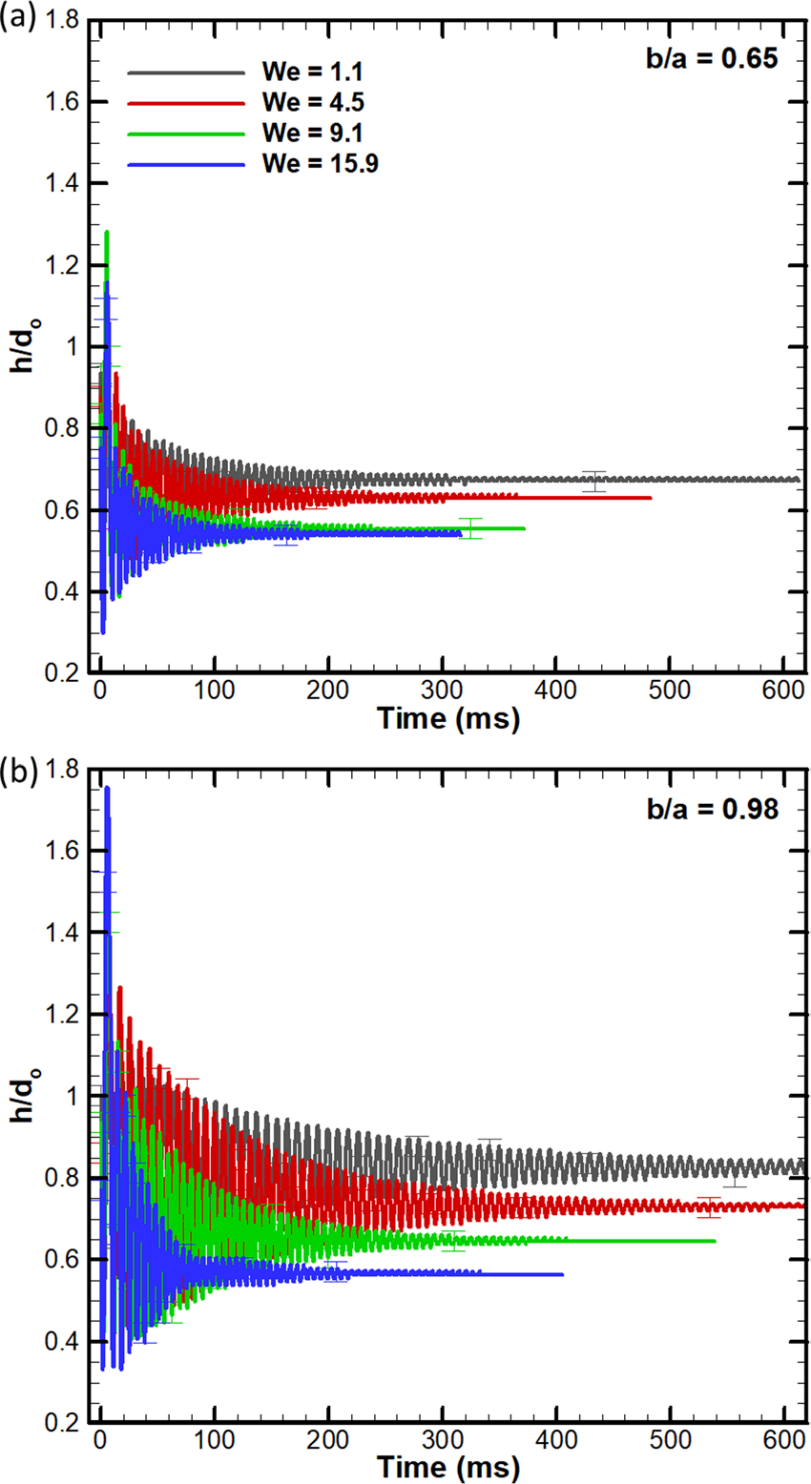
Time-varying instantaneous dimensionless droplet height (*h*/*d*_0_) on a bioinspired surface at different Weber numbers (*We*), (**a**) *b/a* = 0.65 (**b**) *b/a* = 0.98. A plateau signal of *h*/*d*_0_ implies that the droplet has become sessile.

The impact dynamics is further quantified by plotting the time-variation of the dimensionless height (*h*/*d*_*o*_) of the droplet until droplet becomes sessile. Figure 14(a,b) shows *h*/*d*_*o*_ on the surfaces with the smallest and the largest *b/a* (=0.65 and 0.98), respectively at different *We*. The oscillatory response of *h*/*d*_*o*_ is similar to response of an underdamped spring-mass system, in which the oscillation amplitude decays exponentially. Here, the surface tension and viscosity manifest as stiffness force and damping force, respectively. The estimated oscillation frequency (using FFT algorithm) is in the range of 0.12–0.18 mHz and agrees with the prediction of a model^27,28^ ($f \sim \sqrt{\gamma /\rho {d}_{o}^{3}}=0.12$ mHz). The oscillation amplitudes of *h*/*d*_*o*_, plotted in Fig. 14, are larger in case of *b/a* = 0.98 as compared to *b/a* = 0.65 at respective *We*. In addition, the time taken to reach the sessile state is larger in the former than the latter, at respective *We*. As mentioned earlier, this is due to larger kinetic energy available during the recoil in case of *b/a* = 0.98, due to larger hydrophobicity of this surface.

## Discussion

We have carried out an investigation of wetting characteristic and droplet impact dynamics on *Colocasia esculenta* (taro) leaf and bioinspired surfaces based on the morphology of the leaf. We analyzed the morphology of the surface of the leaf using scanning electron microscopy. The measured static, advancing, and receding angles on the leaf are 150°, 153° and 144°, respectively, with an uncertainty of around ±2°. Biomimicking of the leaf surface was carried out using standard photolithography techniques, and we manufactured bioinspired surfaces with hexagonal microcavities of different sizes i.e a honeycomb-like structure. The ratio of inner to the outer radius (*b*/*a*) of the circumscribed circle to the hexagon was varied and different surfaces were manufactured. We found that the contact angle of the bioinspired surface monotonically increases with *b*/*a*. The measured static angle on the surface was in the range of 104° to 148°. Measured static contact angle as a function of *b*/*a* are in good agreement with predictions by a free-energy based model while the droplet is in Cassie-Baxter state.

The measured contact angle hysteresis on the leaf was very small (9°), opposite to that of the bioinspired surfaces. The droplets stick to the bioinspired surface even those with a larger contact angle of 149°. This difference between the two surfaces is attributed to the fact that we were not able to mimic the nanoscale second-tier features on the wall of the hexagonal cavity (Fig. 2c,d) on the bioinspired surface. A second or larger tier helps in increasing hydrophobicity and in improving water-repellency of the surface^12^. The sticking behavior of the bioinspired surface is also opposite to a bioinspired surface with micropillars, reported in previous studies (e.g. Patil *et al*.^13^). We employed a first-order model based on surface tension and gravitational forces to explain this behavior. It was found that a continuous contact line on the former surface is responsible for the sticking behavior, as compared to an engineered surface with micropillars.

Further, we measured droplet impact dynamics to study water repellency characteristics of the leaf and bioinspired surfaces. The droplets bounce on the leaf beyond a critical Weber number (*We*); however, droplets stick to the bioinspired surfaces in all cases of *We*. At larger *We*, we recorded droplet breakup on the surface with larger *b*/*a*. We propose a two-dimensional regime map as function of *We* and *b*/*a*, to demarcate the two regimes namely, *no-breakup* and *breakup*. The droplet assumes Wenzel or partial Wenzel state at larger *We* and *b*/*a*. The latter is a function of *We* and *b*/*a* and the measured angles are closer to the prediction of the free-energy based model.

Overall, the present results provide new fundamental insights on wetting and water repellency characteristics of the taro leaf and bioinspired surfaces based on the first-tier structure on the leaf. The taro leaf shows remarkable superhydrophobic and water repellency properties. The bioinspired surfaces exhibit contact angle on a par with the leaf and could be engineered to achieve desired wettability. Although the bioinspired surfaces show poor water repellency characteristics, yet such surfaces can be employed to design devices for water-harvesting (e.g. Sharma *et al*.^17^), due to their droplet sticking nature.

## Methods

### Experimental details

Leaves of *Colocasia esculenta* were collected at IIT Bombay campus, Mumbai, India (19° 07′43″N, 72° 54′48″E). The samples were carefully chosen and were screened for any damages or dirt deposits. The images of leaves before samples collection are provided in supplementary information. To prepare a sample for measurement, a freshly plucked leaf was washed with running deionized water. Nitrogen was blown gently on the surface of the leaf to remove the residual water. A small part of the leaf (1 cm by 1 cm) was cut and was stuck on a glass slide for the measurements. We used SEM (JSM-7600F, Jeol Inc.) to characterize the microstructures on the surface of the leaf.

#### Contact angle measurement

**Figure 15:**
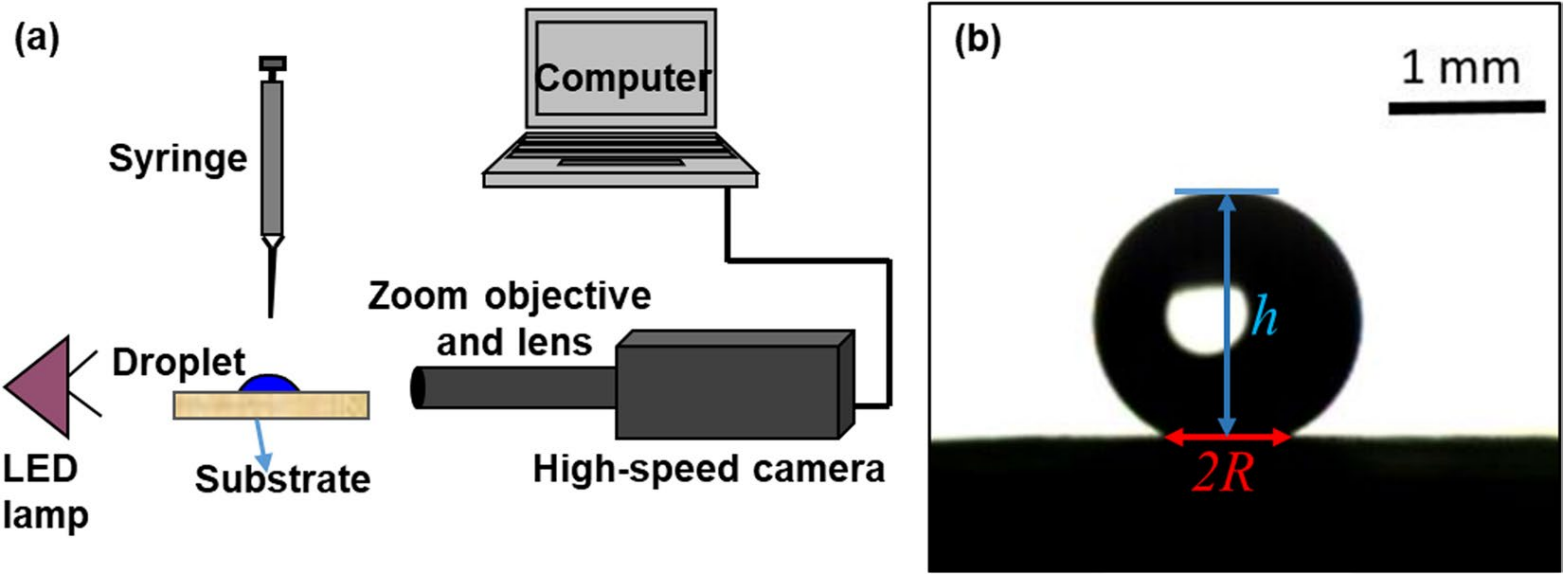
(**a**) Experimental setup used in the present study. (**b**) A typical image obtained by side visualization of a water droplet resting on the surface of the taro leaf or the bioinspired surface. Droplet height (*h*) and wetted diameter (2*R*) are shown on the image.

To measure the static contact angle of a water droplet on the leaf and on the bioinspired surfaces, we gently placed a droplet on the prepared sample using a 31-gauge needle syringe. We recorded the side view of the droplet and analyzed it in an image processing software. The bioinspired surfaces did not exhibit anisotropic wetting i.e., the contact angles recorded in different directions were almost same. A typical image of a water droplet resting on the leaf surface is shown in Fig. 15b. A microliter droplet (less than 3.9 *μ*L) assumes spherical cap shape on the leaf. By measuring the wetted radius (*R*) and height of the droplet (*h*) we calculated the static contact angle (*θ*) of the droplet using the following equation for the spherical cap geometry:

4 $$\theta =2\,{\rm{ta}}{{\rm{n}}}^{-1}\left(\frac{h}{R}\right)$$

To measure advancing (*θ*_*a*_) and receding (*θ*_*r*_) angle, the droplet sliding method was employed. The leaf sample was put on a rotating stage, and a droplet was placed on it gently. The stage was rotated very slowly (1-2°/s) until the droplet started to slide and the droplet dynamics from the side was recorded using a high-speed camera (MotionPro, Y-3 classic) with long-distance working objective (Qioptiq Inc.) at 60 frames per second. *θ*_*a*_ and *θ*_*r*_ were obtained at the onset of the sliding using the image processing software.

#### Fabrication of bioinspired surfaces

The bioinspired surfaces in our study mimicked first-tier structure found on the surface of the leaf. The shape of the first-tier structure resembles a microcavity, with the shape of either hexagon or pentagon. The hexagon was chosen over pentagon as base geometry for the honeycomb structure as the number of hexagons were higher than pentagon in the leaf (more than 70%). They were fabricated using standard photolithography techniques on a silicon wafer. We used the fabrication process described in Refs. ^13,29^ and is briefly described as follows in the following steps:

1. CAD geometry having desired geometrical structure was created in AUTOCAD-R24.0 software. The details of AUTOCAD drawings are provided in the supplementary information.
2. Using a laser writer (LW405, Microtech Inc), the geometrical structure is written over an iron oxide coated mask plate.
3. Standard RCA cleaning of the silicon wafer was carried out to remove contaminants.
4. SU8-2025 was spin-coated on the wafer at the speed of 500 rpm for 10 s and 2300 rpm for 40 s.
5. Coated wafer was prebaked at 65 °C for 3 min and at 95 °C for 8 min.
6. The mask plate was aligned on top of a coated silicon wafer using a single-sided aligner (MJB4, Karl Suss MicroTech Inc) and exposed to ultraviolet radiation.
7. The coated silicon wafer was post-baked at 65 °C for 1 min and at 95 °C for 6 min and was allowed to cool in ambient conditions.
8. The coated wafer was developed using SU-8 photo developer for 5 min and was cleaned using isopropanol.
9. Lastly, the wafer was hard-baked at 120 °C for 10 min and allowed to cool in ambient conditions.

The morphology of the fabricated surfaces was characterized by SEM (JSM-7600F, Jeol Inc.) and optical profilometery (Zeta-20, Zeta Instruments Inc.). The values of the side and thickness of the hexagonal cavity on the surfaces are listed in Table 2. The average depth or height of the hexagon cavities is 29 ± 1 *μ*m.

#### High-speed visualization of the droplet impact dynamics

As shown in a schematic in Fig. 15a, we employed high-speed visualization from the side, as reported in our previous studies^13,14,30^. The optical system comprised of a high-speed camera (MotionPro, Y-3 classic) with long-distance working objective (Qioptiq Inc.). A white LED lamp served as a source of backlight. The working distance, image resolution and pixel resolution for the high-speed camera were 9.5 cm, 600-by-450 and 71 pixels/mm, respectively. The images were recorded at 60 frames per second for the contact angle measurement and 2000 frames per second were used for the droplet impact measurement. Droplet impact velocities *v* = 0.07, 0.22, 0.43, 0.62 and 0.82 m/s were achieved by dripping the droplet from the height of 0.3, 2.46, 9.424, 19.59 and 34.27 mm, respectively. The corresponding Weber number (*We* = *ρv*^2^*d*_*o*_/*γ*, where *ρ*, *v*, *d*_*o*_ and *γ* are density, impact velocity of the droplet, initial diameter before impact and surface tension, respectively) to these impact velocities are 0.1, 1.1, 4.5, 9.1 and 15.9, respectively.

### Theoretical model

We designed textured surfaces with hexagon as base structure, using a model reported by Patankar^22^. The model predicts static contact angle in Cassie-Baxter and Wenzel state. Figure 4 shows the base geometry of the honeycomb structure and its geometrical parameters, needed as input in the model. We obtained the expressions of the contact angle in Cassie-Baxter and Wenzel state for the present study using Patankar’s model^22^ and these are described in the following subsections.

#### Cassie-Baxter state

The static contact angle on a textured surface in Cassie state is as follows^31,32^:

5 $$cos{\theta }^{c}={\phi }_{s}(cos{\theta }_{e}+1)-1$$

where *ϕ*_*s*_ is area fraction of the liquid-solid contact. *θ*_*e*_ is the static contact angle of the liquid drop on the flat surface and ${\theta }^{c}$ is contact angle in Cassie state. In case of Cassie state, droplet sits on the top surface of honeycomb structure, so the area of honeycomb surface (solid) which will be in contact with droplet (liquid) will be the annular area (*A*_*ls*_) of unit cell as shown in Fig. 4, which is as follows:

6 $${A}_{ls}=\frac{3\sqrt{3}}{2}({a}^{2}-{b}^{2})$$

The total area of the unit cell (*A*_*t*_) is given by:

7 $${A}_{t}=\frac{3\sqrt{3}}{2}{a}^{2}$$

Hence the area fraction of the liquid-solid contact (*ϕ*_*s*_) is expressed by using Eq. 6 and Eq. 7 as follows:

8 $${\phi }_{s}=\frac{{A}_{ls}}{{A}_{t}}=\frac{\frac{3\sqrt{3}}{2}({a}^{2}-{b}^{2})}{\frac{3\sqrt{3}}{2}{a}^{2}}=1-\frac{{b}^{2}}{{a}^{2}}$$

Substituting the expression of *ϕ*_*s*_ obtained in Eq. 8 in Eq. 5, the expression for contact angle on a honeycomb surface in the Cassie state is given by:

9 $$cos{\theta }^{c}=(1-\frac{{b}^{2}}{{a}^{2}})(cos{\theta }_{e}+1)-1$$

#### Wenzel state

The static contact angle on a textured surface in Wenzel state is as follows^32,33^:

10 $$cos{\theta }^{w}=rcos{\theta }_{e}$$

where *r* is the ratio of the actual area of liquid-solid contact to the projected area of droplet surface contact on the horizontal plane. The actual area (*A*_*w*_) of solid in contact with liquid is area *A*_*t*_ plus the area of six inner wall of the honeycomb microtexture, which can be expressed as follows:

11 $${A}_{w}=\frac{3\sqrt{3}}{2}{a}^{2}+6bH$$

where *H* is the height of the honeycomb walls and the projected area of droplet surface contact will be same as *A*_*t*_. Therefore, *r* is expressed as follows:

12 $$r=\frac{{A}_{w}}{{A}_{t}}=\frac{\frac{3\sqrt{3}}{2}{a}^{2}+6bH}{\frac{3\sqrt{3}}{2}{a}^{2}}=1+\frac{4(b/a)}{\sqrt{3}(a/H)}$$

Therefore, on substituting the expression of *r* obtained in Eq. 12 in Eq. 10, the final expression of the static contact angle on a honeycomb surface in the Wenzel state is as follows:

13 $$cos{\theta }^{w}=(1+\frac{4(b/a)}{\sqrt{3}(a/H)})cos{\theta }_{e}$$

## Supplementary information

Supplementary Information 1.

Supplementary Information 2.

Supplementary Information 3.

Supplementary Information 4.

Supplementary Information 5.

Supplementary Information 6.

Supplementary Information 7.

Supplementary Information 8.

Supplementary Information 9.

Supplementary Information 10.

Supplementary Information 11.

Supplementary Information 12.

Supplementary Information 13.
