# Supplementary material for: Wetting characteristics of Colocasia esculenta (Taro) leaf and a bioinspired surface thereof
Source: Sci Rep. 2020 Jan 22;10:935. doi: 10.1038/s41598-020-57410-2 (PMC6976613; doi:10.1038/s41598-020-57410-2)
Supplement: Supplementary file 13 — Supplementary Information 13. [file 41598_2020_57410_MOESM13_ESM.pdf]

Supporting Information: Wetting characteristics of *Colocasia esculenta* (Taro) leaf and a bioinspired surface thereof

Manish Kumar, Rajneesh Bhardwaj*

Department of Mechanical Engineering,
Indian Institute of Technology Bombay, Mumbai 400076, India.
*Corresponding author (email: rajneesh.bhardwaj@iitb.ac.in)

S1 Photographs of Taro leaves

We provide additional images of the Taro leaves in Figs. S1 and S2. Optical microscopic images of two different size of the leaf are provided in Fig. S3.

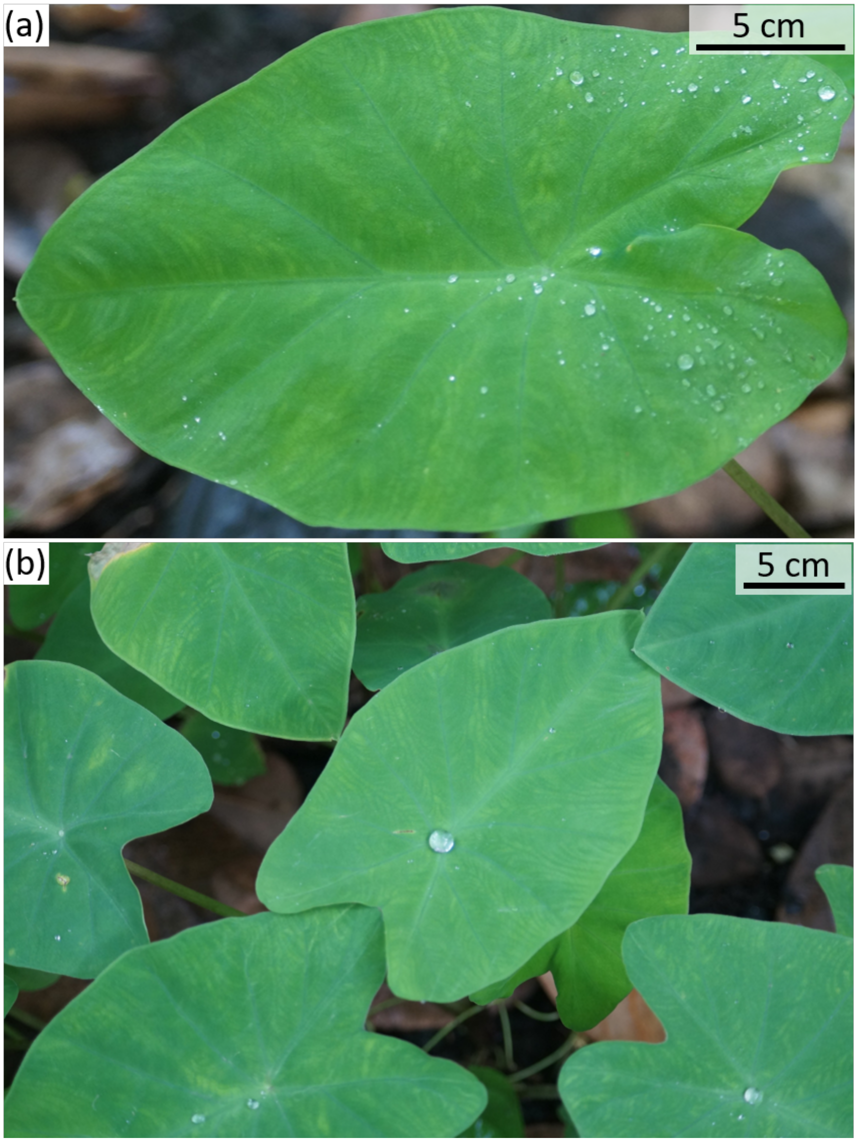

Figure S1: Taro Leaves in Garden

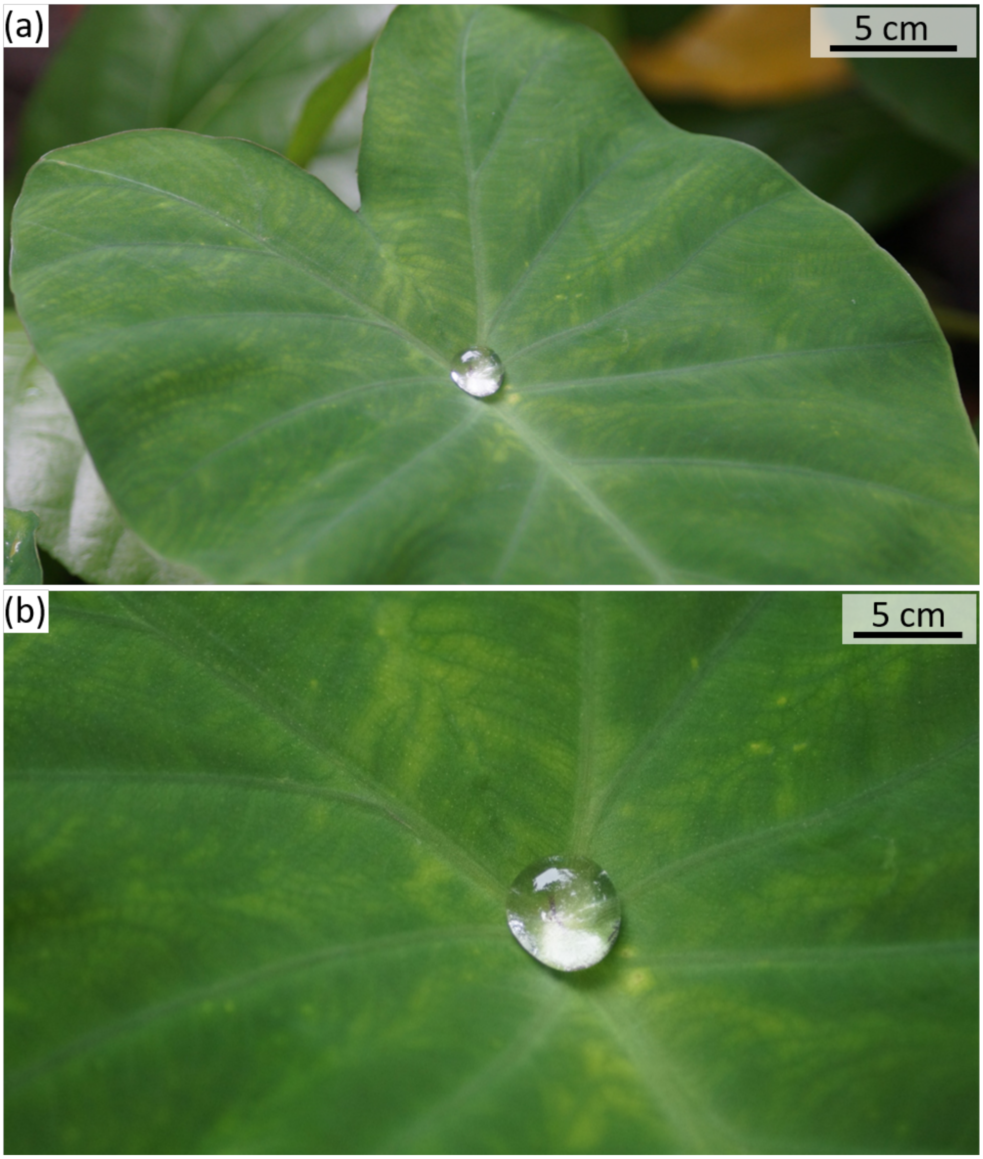

Figure S2: Water collected on the leaf showing high contact angle.

S2 Optical microscopic images of the leaf

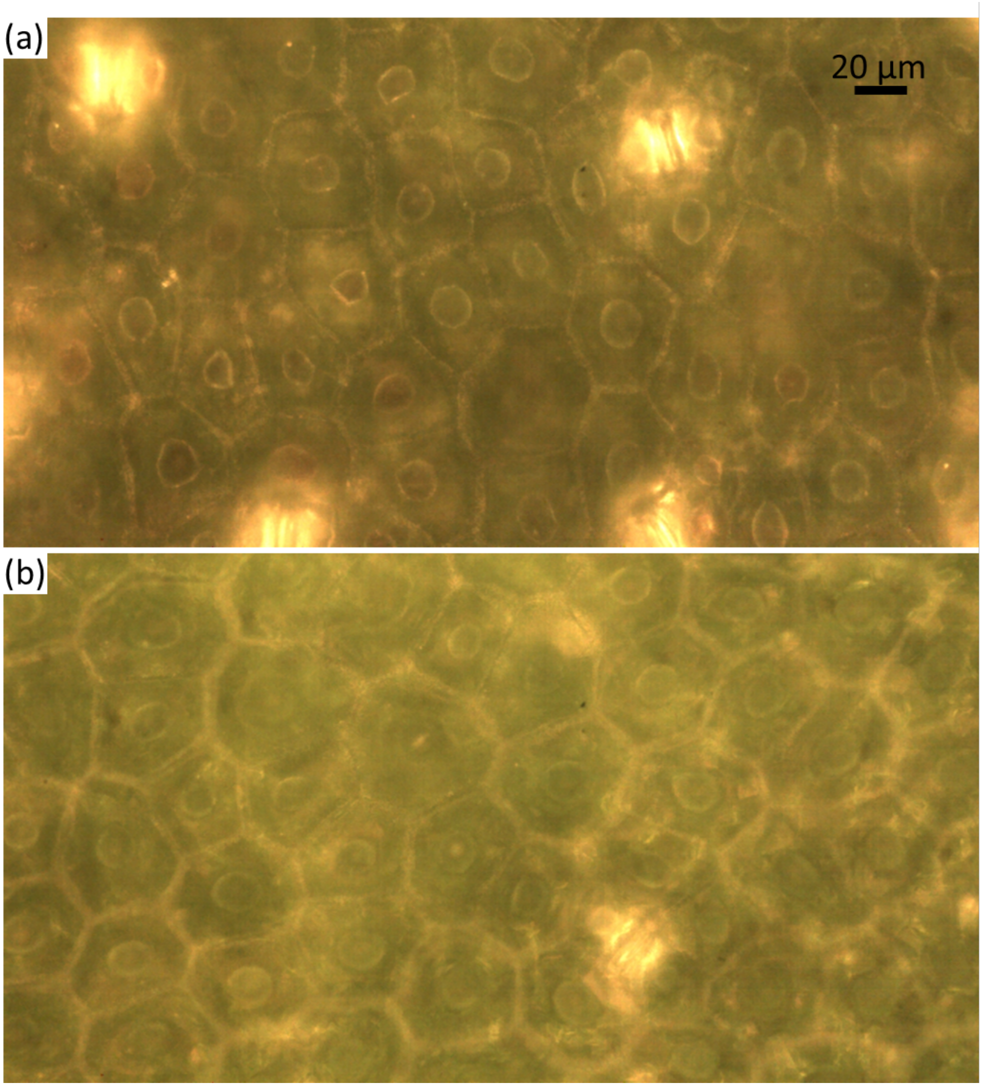

Figure S3: Optical microscopy images of leaf captured at 400X magnification. (a) Large size or fully grown leaf, (b) Small size leaf. Glowing part in the images is stomata of the leaf.

S3 SEM of leaf

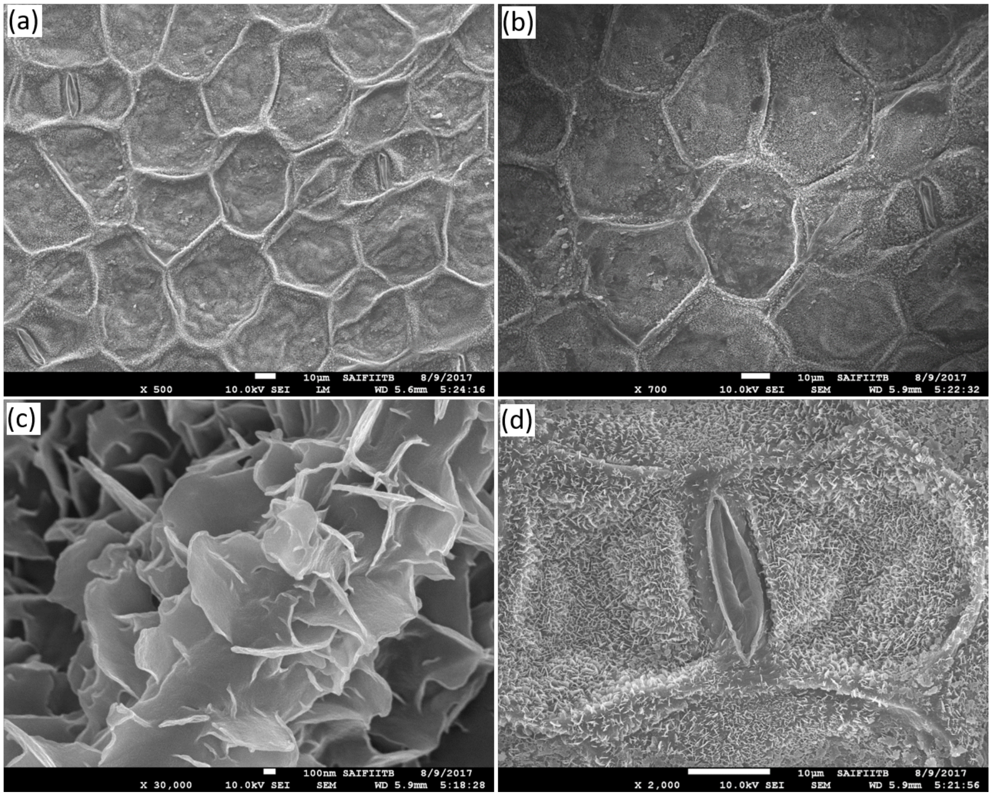

Figure S4: (a) SEM at 500X (b) SEM at 700X (c) nano-structures on leaf (d) Stomata of leaf

S4 Wetting characteristics on rear side of the leaf

To quantify the wetting characteristics on the back side of the leaf, we measured the contact angle, advancing angle and receding angle of a water droplet on the leaf. These measurements are similar to those on the top side of the leaf. We repeated the experiments four times at different location of the same leaf and on different leaves for different volumes of the droplet. These measurements are listed in Table S1.

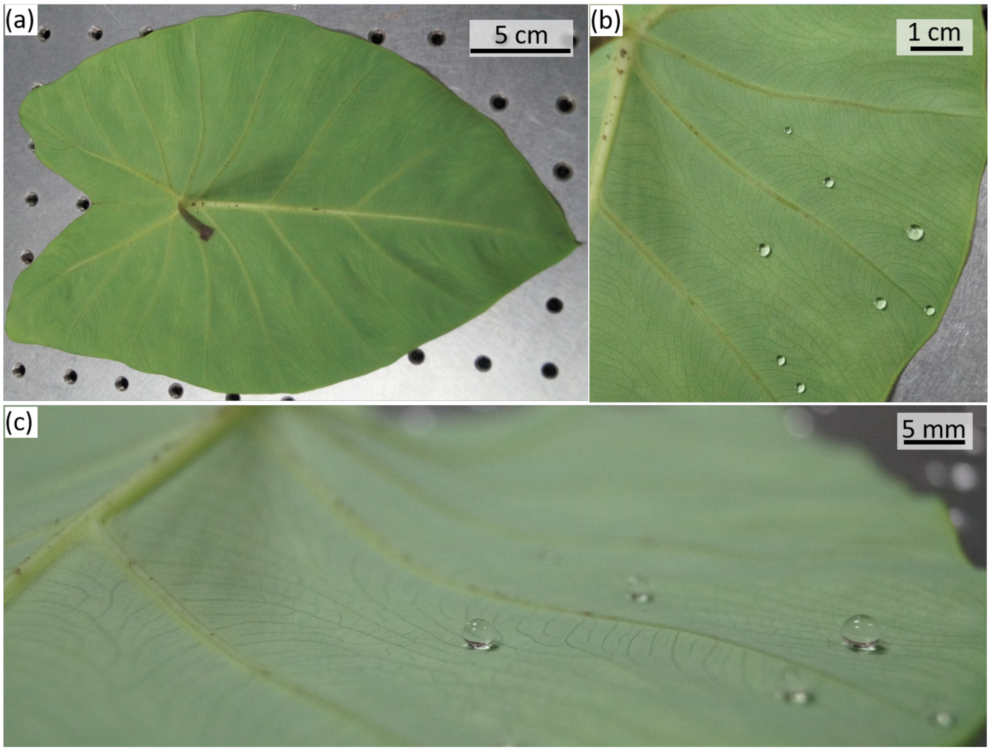

Figure S5: (a) Back side of the leaf (b) Top view of the back side with sessile droplets (c) Tilt view of the leaf with sessile droplets on the back side.

Volume (μL)	Static contact angle ($^\circ$)	Advancing contact angle ($^\circ$)	Receding contact angle ($^\circ$)
2.7	151.2	154	146
2.5	152.2	153	145
2.3	150.9	154	142
3.1	149.0	152	143

Table S1: Measurements of static contact angle, advancing contact angle and receding contact angle on the back side of the leaf

S5 CAD geometry of bioinspired surface

CAD geometry with desired geometrical structure (Figure S6) was created as input to laser writer.

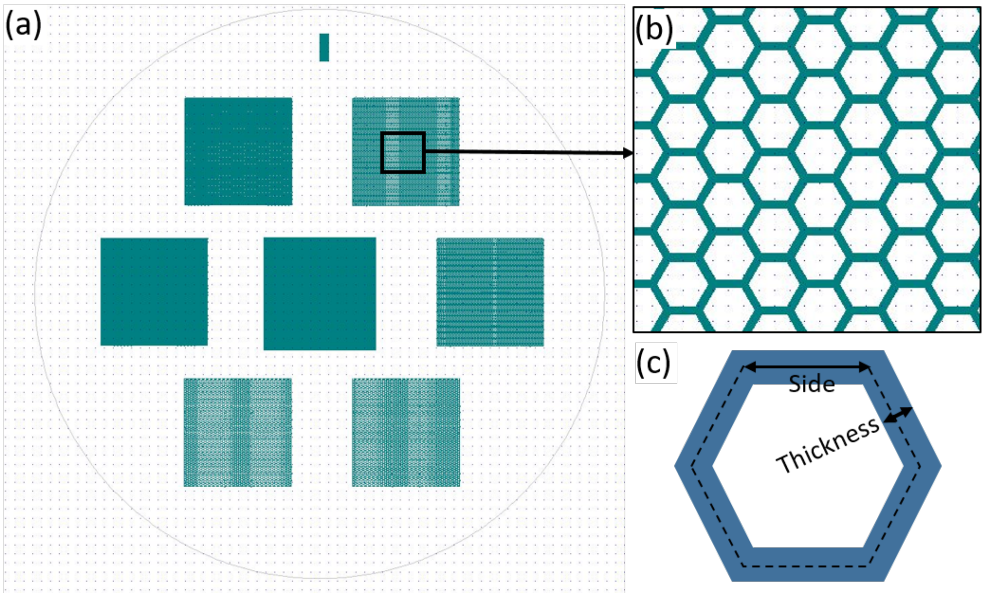

Figure S6: (a) Snapshot of CAD geometry. (b) Zoomed-in view of honeycomb structures. (c) Definition of parameters side and thickness of honeycomb structure. AUTOCAD-2018 was used to create the CAD model.

S6 Microscopic images of bioinspired surface

Figure S7 shows the twelve microscopic images of fabricated surfaces having different thickness and side of hexagon. The label at the center of the each image indicates the contact angle measured on respective surface.

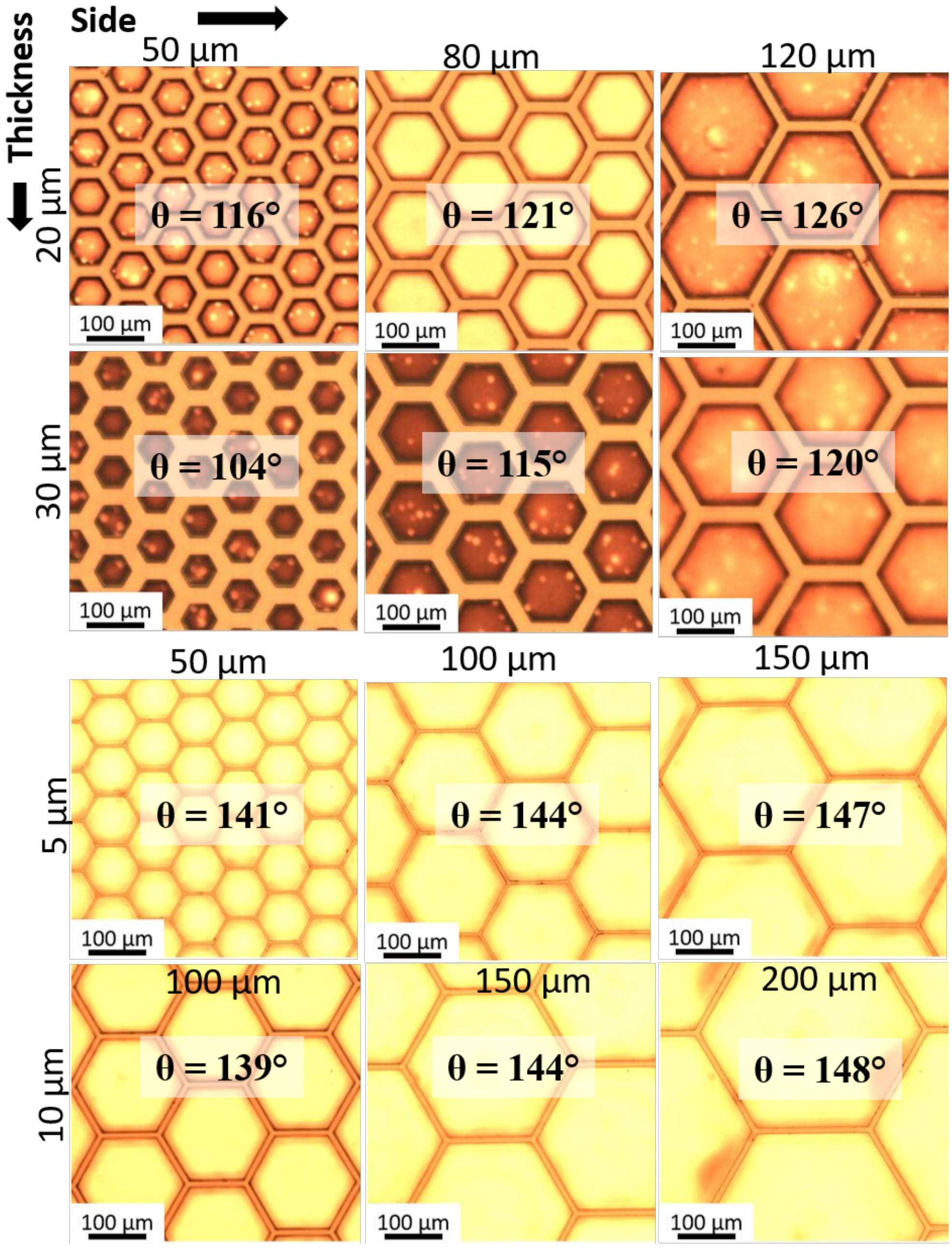

Figure S7: Microscope images of fabricated surfaces taken at 100x magnification. Parameters side, thickness and contact angle (θ) are indicated for each image.
